# Supplementary material for: In-Depth Investigation of Low-Abundance Proteins in Matured and Filling Stages Seeds of Glycine max Employing a Combination of Protamine Sulfate Precipitation and TMT-Based Quantitative Proteomic Analysis
Source: Cells. 2020 Jun 22;9(6):1517. doi: 10.3390/cells9061517 (PMC7349688; doi:10.3390/cells9061517)
Supplement: Supplementary file 1 [file cells-09-01517-s001.zip › Supplementary figures.pptx]

## Slide 1
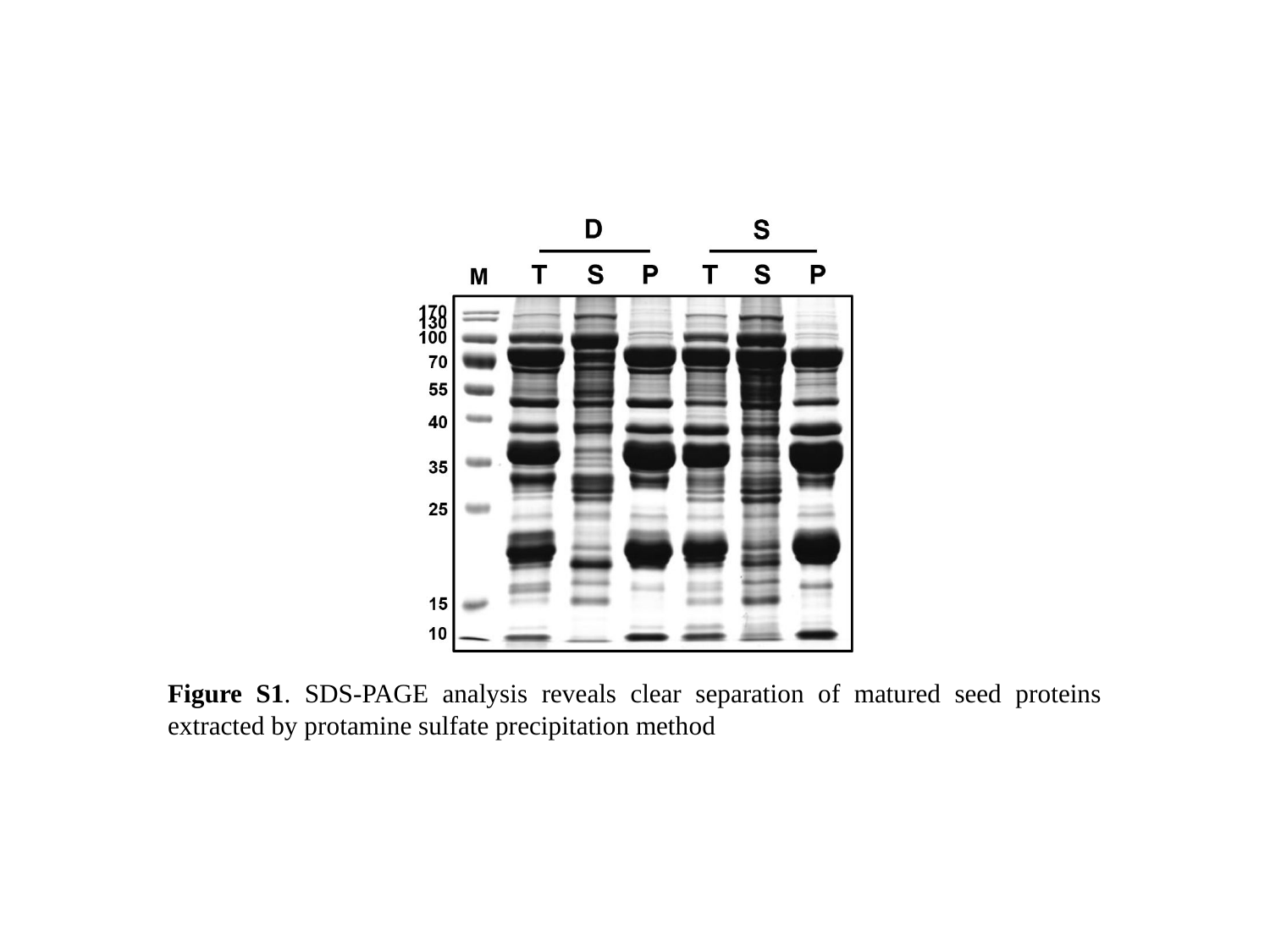

Figure S1. SDS-PAGE analysis reveals clear separation of matured seed proteins extracted by protamine sulfate precipitation method

## Slide 2
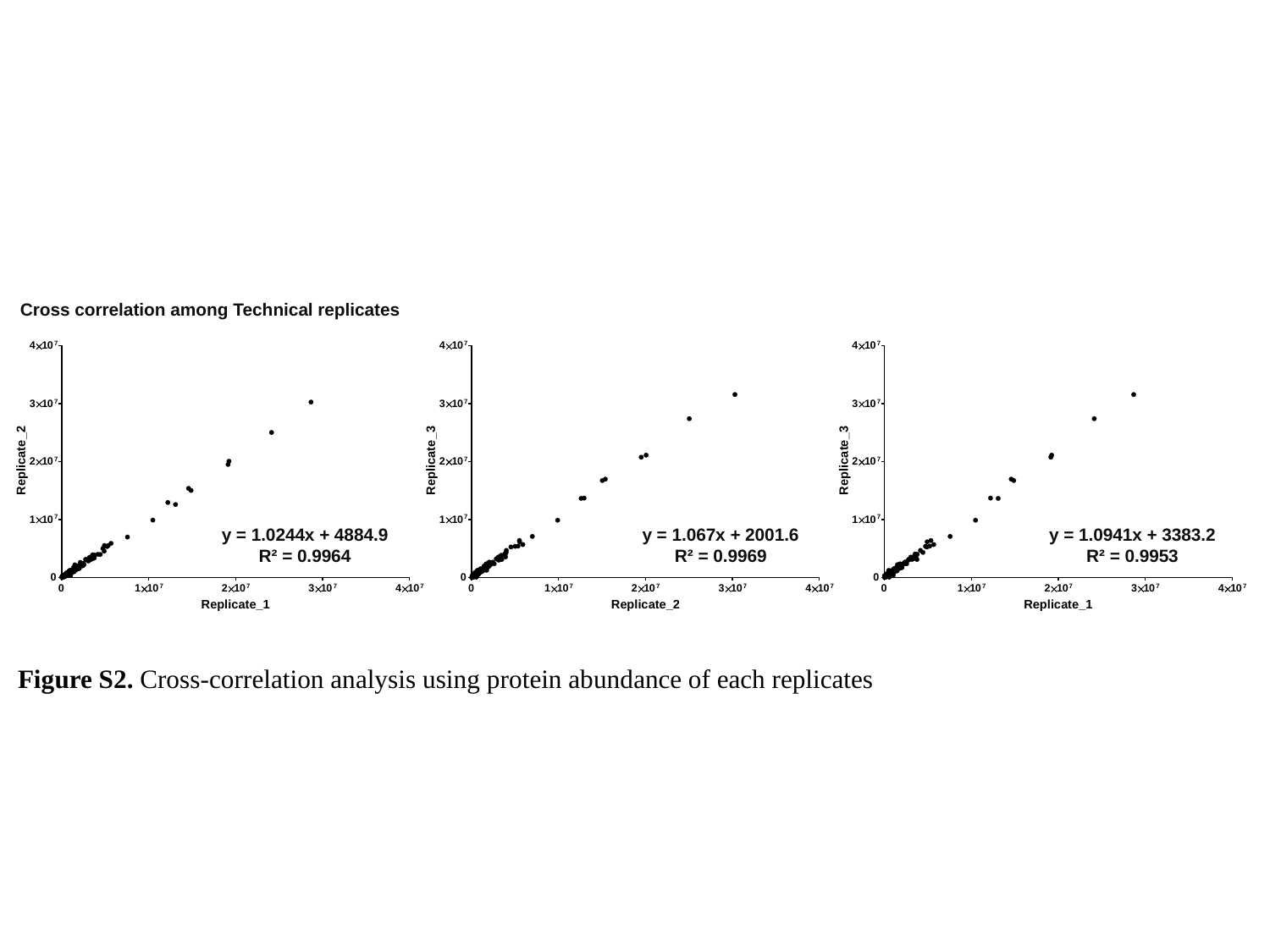

Cross correlation among Technical replicates
y = 1.0244x + 4884.9
R² = 0.9964
y = 1.067x + 2001.6
R² = 0.9969
y = 1.0941x + 3383.2
R² = 0.9953
Figure S2. Cross-correlation analysis using protein abundance of each replicates

## Slide 3
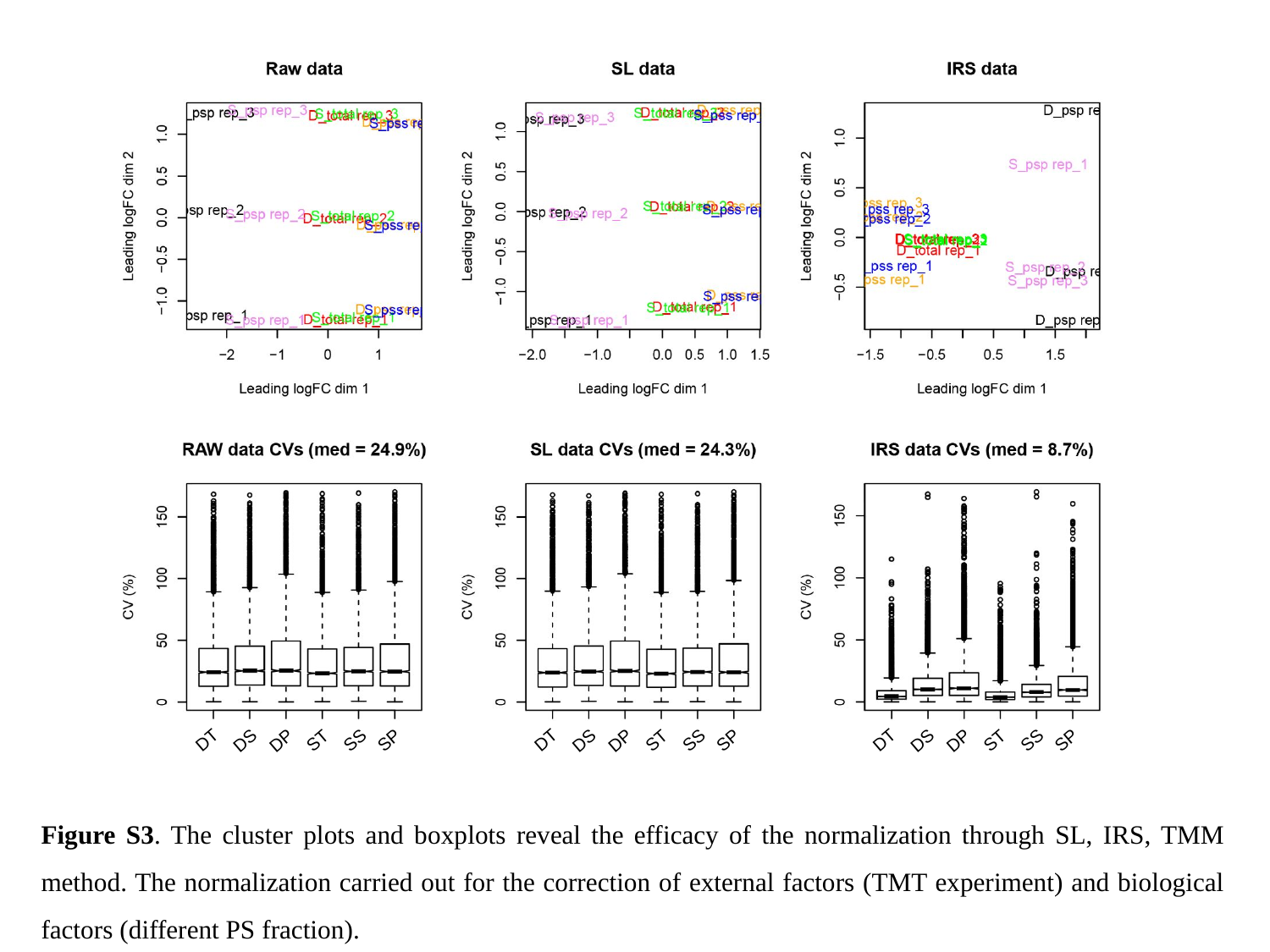

DT
DS
DP
ST
SS
SP
DT
DS
DP
ST
SS
SP
DT
DS
DP
ST
SS
SP
Figure S3. The cluster plots and boxplots reveal the efficacy of the normalization through SL, IRS, TMM method. The normalization carried out for the correction of external factors (TMT experiment) and biological factors (different PS fraction).

## Slide 4
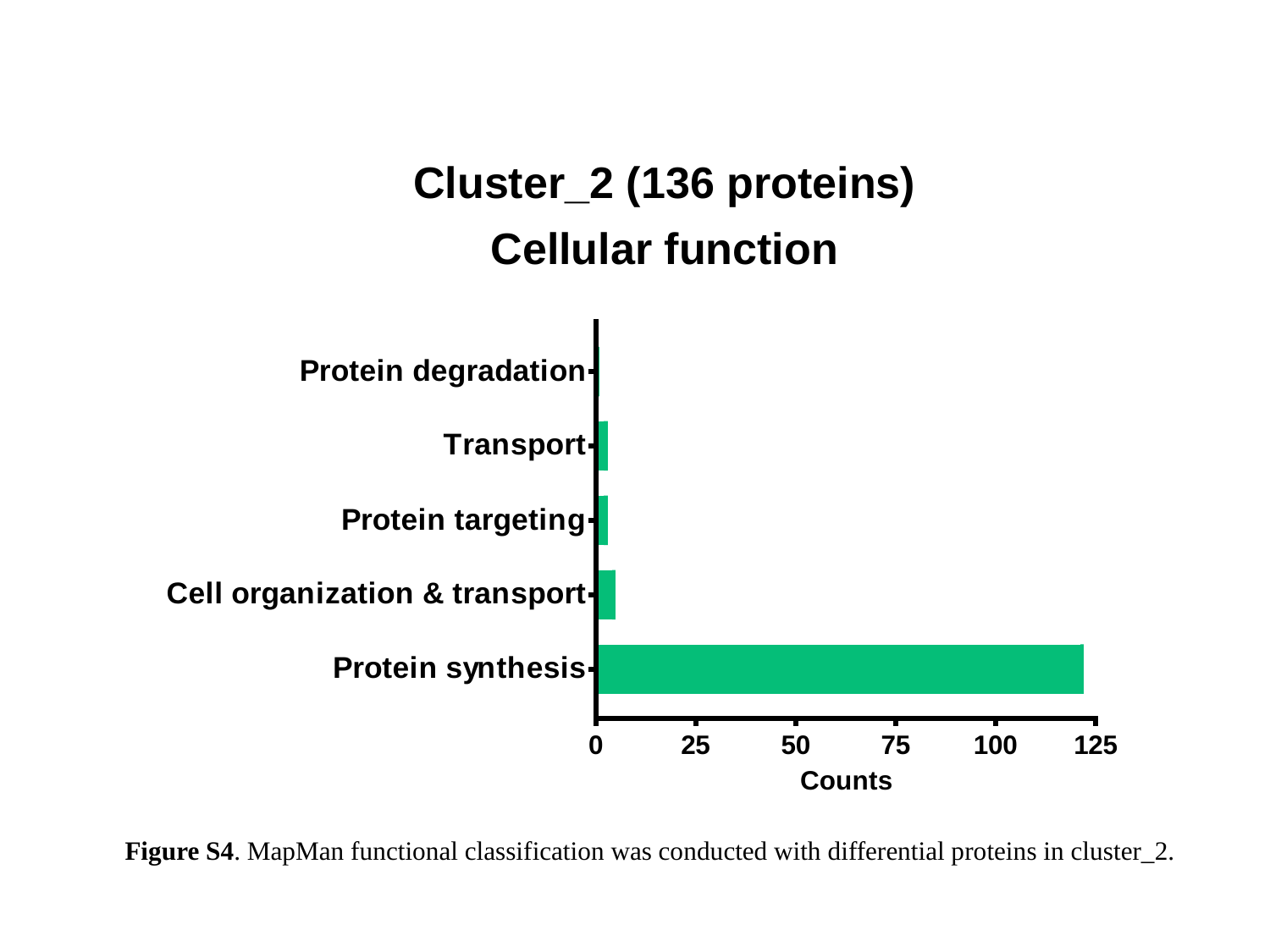

Cluster_2 (136 proteins)
Cellular function
Figure S4. MapMan functional classification was conducted with differential proteins in cluster_2.

## Slide 5
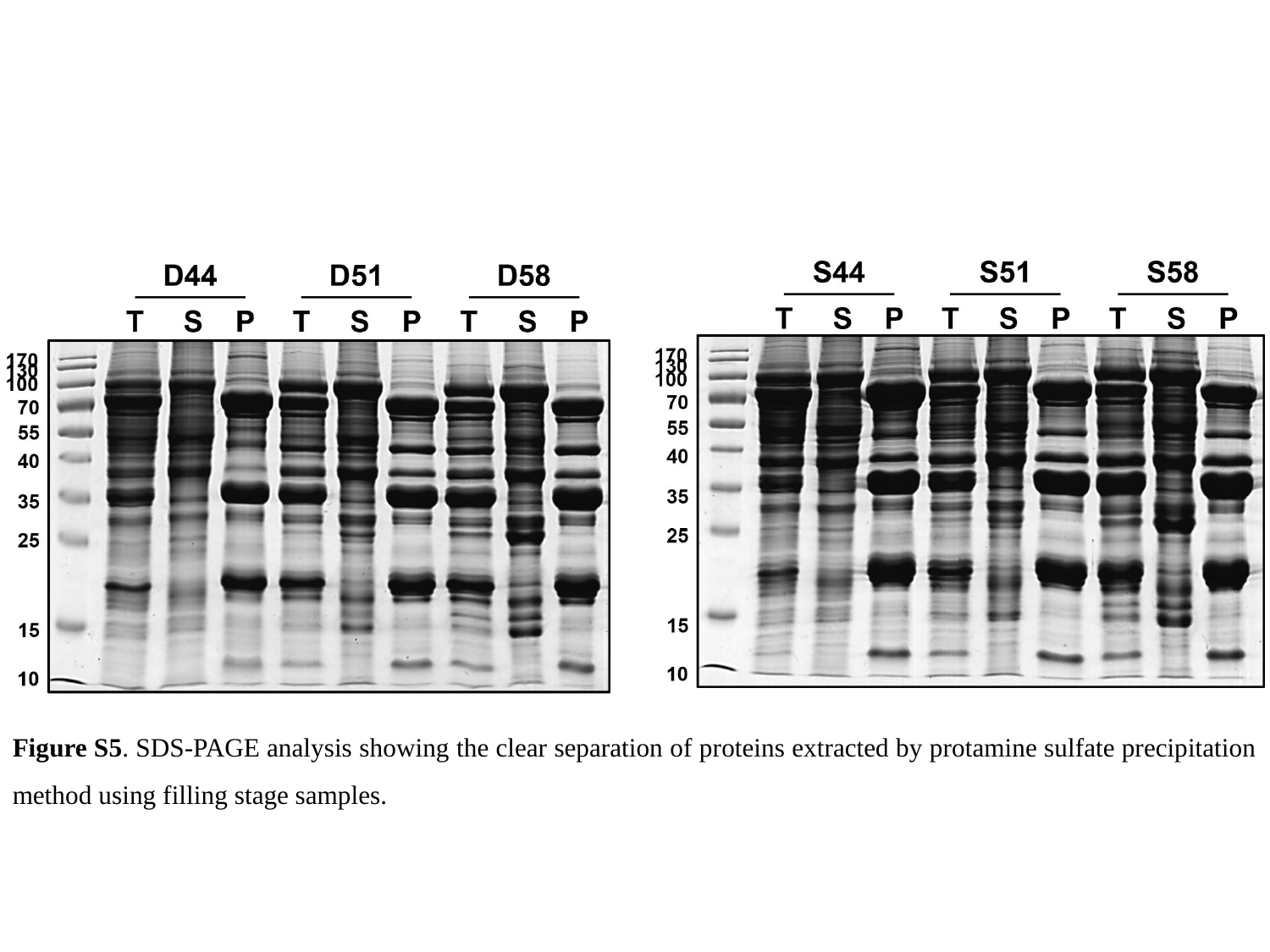

Figure S5. SDS-PAGE analysis showing the clear separation of proteins extracted by protamine sulfate precipitation method using filling stage samples.

## Slide 6
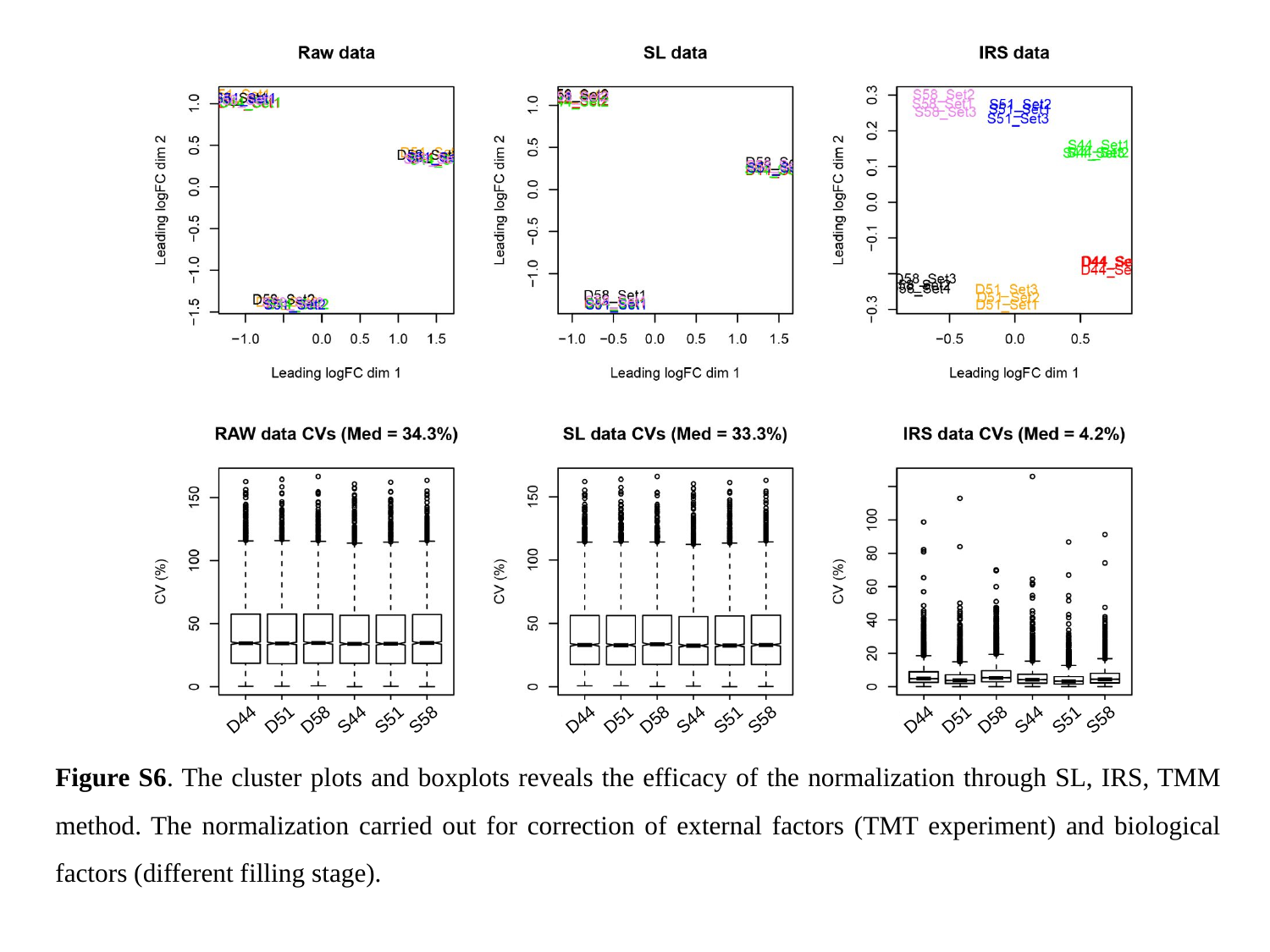

D44
D51
D58
S44
S51
S58
D44
D51
D58
S44
S51
S58
D44
D51
D58
S44
S51
S58
Figure S6. The cluster plots and boxplots reveals the efficacy of the normalization through SL, IRS, TMM method. The normalization carried out for correction of external factors (TMT experiment) and biological factors (different filling stage).

## Slide 7
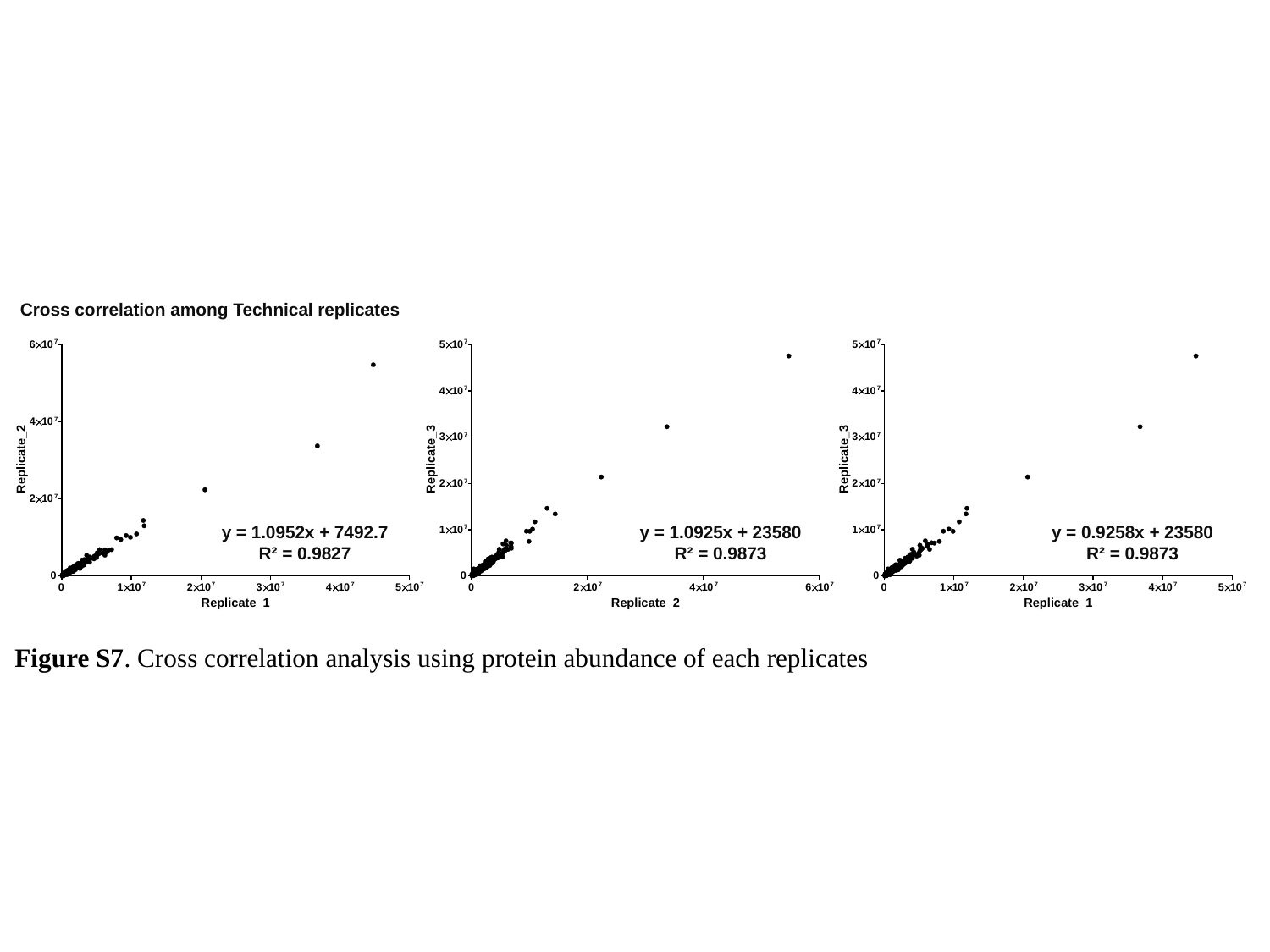

Cross correlation among Technical replicates
y = 1.0952x + 7492.7
R² = 0.9827
y = 1.0925x + 23580
R² = 0.9873
y = 0.9258x + 23580
R² = 0.9873
Figure S7. Cross correlation analysis using protein abundance of each replicates

## Slide 8
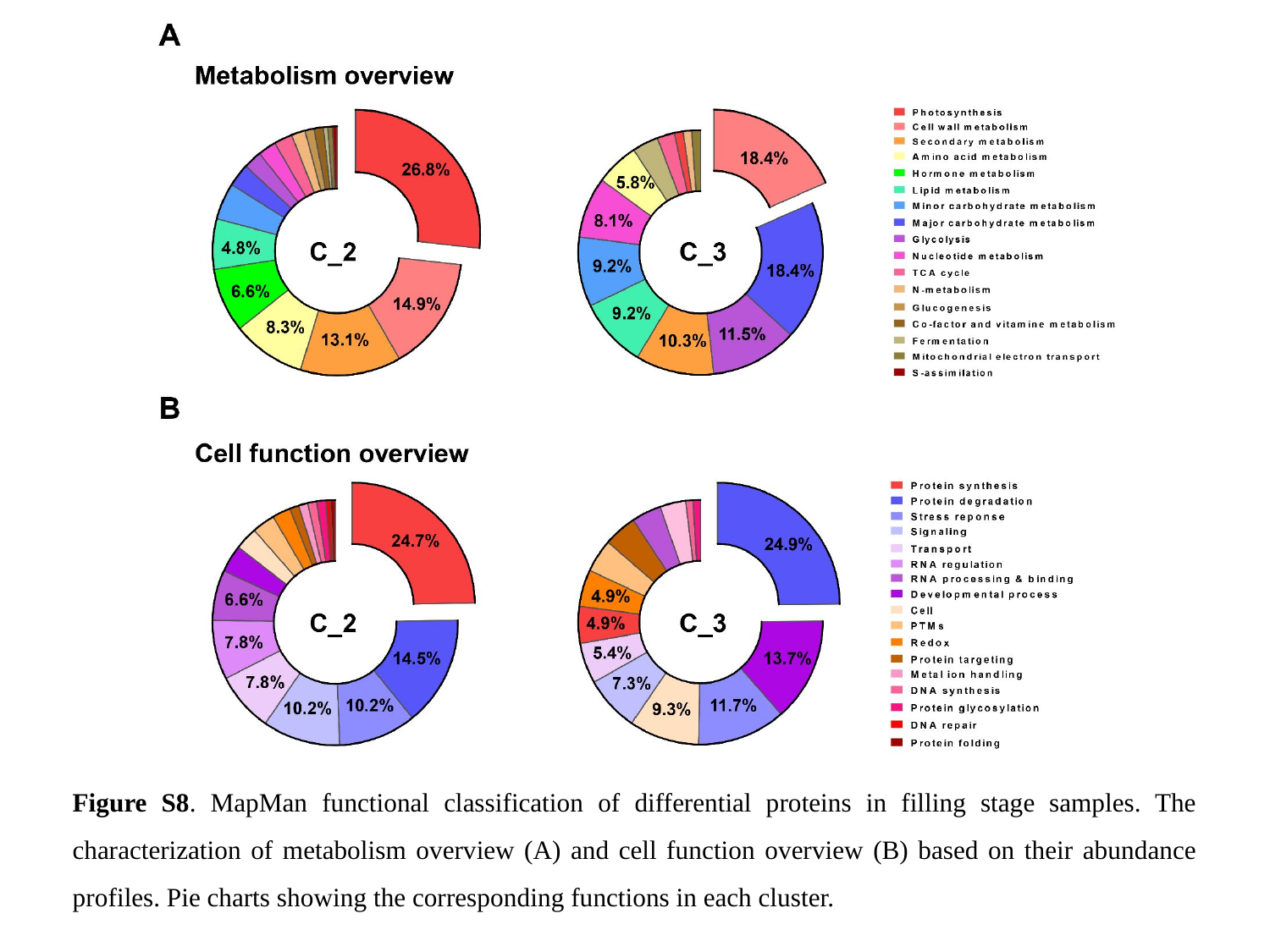

Figure S8. MapMan functional classification of differential proteins in filling stage samples. The characterization of metabolism overview (A) and cell function overview (B) based on their abundance profiles. Pie charts showing the corresponding functions in each cluster.
